# Retraction: Cui S‐Z, Lei Z‐Y, Guan T–P, et al. Targeting USP1‐dependent KDM4A protein stability as a potential prostate cancer therapy. Cancer Science. 2020; 111: 1567–1581. https://doi.org/10.1111/cas.14375

**DOI:** 10.1111/cas.16018

**Published:** 2023-12-11

The above article, published online on 4 March 2020 on Wiley Online Library (wileyonlinelibrary.com), has been retracted by agreement between the journal's Editor‐in‐Chief, Masanori Hatakeyama, the Japanese Cancer Association and John Wiley & Sons Australia, Ltd following concerns raised by a third party about figures within the article. During the journal's investigation into the concerns raised, the authors were not able to provide comprehensive original data for the relevant figures. Accordingly, the editors consider that the results in the published article are unreliable and do not sufficiently support the conclusions. The following authors of the article have stated that they were unaware of its publication: Shu‐Zhong Cui, Zi‐Ying Lei, Tian‐Pei Guan, Hao‐Wu Jiang.

