# Correction

**DOI:** 10.1111/cas.16189

**Published:** 2024-06-02

Correction to “Retraction: Cui S‐Z, Lei Z‐Y, Guan T‐P, et al. Targeting USP1‐dependent KDM4A protein stability as a potential prostate cancer therapy. Cancer Sci. 2020;111:1567–1581. https://doi.org/10.1111/cas.14375.”

The following retraction, published online on 11 December 2023 in Wiley Online Library {https://doi.org/10.1111/cas.16018} has been amended, and the online version has been revised to reflect the correction. The following additional authors have stated they were not aware of the article's publication: Ling‐Ling Fan, Xin‐Yan Geng, De‐Xue Fu.

